# Prevalence and Determinants of Diabetic Foot Ulcers and Lower Extremity Amputations in Three Selected Tertiary Hospitals in Ghana

**DOI:** 10.1155/2019/7132861

**Published:** 2019-02-11

**Authors:** Ambrose Atosona, Christopher Larbie

**Affiliations:** ^1^Department of Biochemistry and Biotechnology, Kwame Nkrumah University of Science and Technology, Kumasi, Ghana; ^2^Department of Nutritional Sciences, University for Development Studies, Tamale Campus, Ghana

## Abstract

**Background:**

The occurrence and complications of diabetes are increasing worldwide. This study examined the prevalence and determinants of diabetic foot ulcers and lower extremity amputations in three selected tertiary hospitals in Ghana.

**Methods:**

A cross-sectional multicenter study involving 100 subjects was carried out. Subjects were selected through simple random sampling from three selected tertiary hospitals in Ghana. A structured questionnaire was used to document information on sociodemographic, medical history, lifestyle, and physical characteristics of subjects. Foot ulcers and lower extremity amputations were also investigated. Total cholesterol, triglycerides, low-density lipoproteins, high-density lipoproteins, serum urea, serum creatinine, and estimated glomerular filtration rate of subjects were assessed. Data analysis was done using SPSS version 22.

**Results:**

The study revealed that 31% and 69% were males and females, respectively, with a mean age of 53.8 ± 13.8 years. Among the patients, 11% had diabetic foot ulcers whilst 3% had lower extremity amputations. In the multivariate binary logistic regression analysis, previous history of foot ulcers (OR = 40.4, 95% CI = 5.5-299.9) and foot deformities (OR = 14.4, 95% CI = 1.3-161.2) were identified as independent predictors of diabetic foot ulcers. Foot deformity (*p* = 0.043) and serum urea (*p* = 0.002) were associated with diabetic lower extremity amputations in the univariate analysis.

**Conclusion:**

This study showed that the prevalences of diabetic foot ulcers and lower extremity amputations are high among diabetes patients. Foot deformities and previous history of foot ulcers are determinants of diabetic foot ulcers. Foot deformity and serum urea are associated with diabetic lower extremity amputations.

## 1. Introduction

Global prevalence of diabetes is high and still on the rise [1]. The prevalences in the world, Africa, and Ghana stand at 8.8%, 3.2%, and 3.6%, respectively [1, 2]. An increase in the prevalence of diabetes is accompanied by an increase in its complications such as foot ulcers and lower extremity amputations, in that, the lifetime risk of a person with diabetes developing a foot ulcer is 25% [3]. The risk for lower extremity amputation is 15 to 40 times higher in people with diabetes than people without diabetes [4].

The complications of diabetes result in reduced quality of life, incapacity, and death [5]. With regard to diabetic foot ulcers, 12% of all hospitalized diabetic patients in Africa have foot ulceration [6]. Research indicates that diabetes patients with foot ulcers encounter stigma, loss of social role, social isolation, and unemployment [7]. Diabetic foot ulcer is a costly and debilitative disease with severe consequences in diabetic patients [8]. Also, mortality after lower extremity amputations in diabetes patients varies from 39% to 80% at 5 years [9–12]. More than half of all nontraumatic lower limb amputations are due to diabetes [13]. Limb amputation causes distortion of body image, increase in dependency, loss of productivity, and increase in costs of treating diabetic foot ulcers [14].

However, the prevalence, risk factors, and predictors of foot ulcers and lower extremity amputations among type 1 and type 2 diabetics have not been investigated in Ghana despite the burden of these complications. Furthermore, period prevalence of diabetic ulcers and amputations vary among countries in hospital settings, in that, diabetic neuropathy, the main cause of these complications, varies widely from country to country depending on the methodology used [6]. For instance, lower extremity amputation varies from 1.5 to 7% (6) and foot ulcers vary from 4-19% [15]. For comparative reasons, it is critical to conduct similar studies in Ghana. Based on the gaps observed above, this study is aimed at defining the extent, the associated risk factors, and predictors of foot ulcers and lower extremity amputations among type 1 and type 2 diabetics in Ghana to help improve preventive strategies to lessen the burden of these diabetes-related complications.

## 2. Materials and Methods

The present paper and that of Atosona et al. [16] are derived from the same research project, hence used similar methodology. Though both papers are derived from the same study, they differ in content and focus as the previous paper [16] focused on sexual dysfunction among diabetics. A cross-sectional multicenter study, involving 100 diabetics randomly selected from the outpatient diabetes clinics of three tertiary hospitals namely Korle Bu Teaching Hospital, Komfo Anokye Teaching Hospital, and Tamale Teaching Hospital, was carried out from June to July 2015.

Subjects for the study were diabetics who were booked and attended the diabetes clinic on a particular clinic day. Subjects diagnosed with diabetes in line with international standards (WHO) (fasting plasma glucose greater than or equal to 7.0 mmol/L and/or 2 hours postprandial plasma glucose or casual plasma glucose greater than or equal to 11.1 mmol/L), under a diabetic diet or antidiabetic drug treatment for at least 1 year, ≥18 years old, and agreed to partake in the study were eligible. Those that were very ill or pregnant were exempted from the study. Subjects who met the inclusion criteria were selected through simple random sampling. The subjects were given random numbers written on pieces of paper, put in a bowl, mixed, and handpicked one at a time with replacement until the required sample was reached.

The Cochran formula [17, 18] was employed in computing the sample size:

(1) $$\begin{array}{c} n=\frac{Z^{2}p\left(1-p\right)}{e^{2}}, \end{array}$$

where *n* is the sample size, *z* is the value for the selected confidence level (usually 1.96 for 95% confidence level), *e* is the level of precision, and *p* is the proportion of an attribute (diabetic foot ulcers) present in the population, which is 3.8% [19] in type 2 diabetics in Ghana. With a desired confidence level of 95% and ±5% precision, a sample size of 56 was obtained.

The sample size was increased to 100 to give better estimate of the population and minimize the effect of outliers. Of the total sample, 33 diabetics were selected from Komfo Anokye Teaching Hospital, 33 from Tamale Teaching Hospital, and 34 from Korle Bu Teaching Hospital.

Sociodemographic characteristics (sex, age, ethnicity, marital status, religion, level of education, and occupation), medical history (duration of diabetes, diabetic diet, diabetes medications, history of poor vision, and previous history of foot ulcers or lower extremity amputations), lifestyle variables (smoking and alcohol intake), and physical characteristics (foot ulcers, lower extremity amputations, foot deformities, BMI, and blood pressure) were documented with a pretested questionnaire. Triglycerides (TG), total cholesterol (TC), high-density lipoproteins (HDL-C), low-density lipoproteins (LDL-C), serum urea, serum creatinine, and estimated glomerular filtration (eGFR) of subjects were also assessed.

According to the International Consensus on Diabetic Foot, a foot ulcer is defined as a full-thickness wound below the ankle in a diabetic patient, irrespective of duration [20]. The operational definition of lower extremity amputation used in this study is the resection of a segment of a lower limb through a bone [21]. In this study, structural abnormalities of the foot such as hammer toes, mallet toes, claw toes, hallux valgus, prominent metatarsal heads, and residuals of neuro-osteoarthropathy, amputations, or other foot surgeries were observed [22]. Results were confirmed with the medical records of the subjects or the consultant physician on duty during the physical examination and were reported as present or absent without further description. The body mass index (BMI) was used to assess obesity. A microtoise (Seca, Germany) was used to measure height (m) without shoes whilst a uniscale (Seca, Germany) was used to measure weight (kg) in light clothing. BMI was computed by dividing the weight (kg) by the square of the height (m^2^) and was classified as underweight (<18.5 kg/m^2^), normal (18.5-24.99 kg/m^2^), overweight (25-29.99 kg/m^2^), and obese (≥30 kg/m^2^). A digital sphygmomanometer (Omron, Japan) was used to measure blood pressure. High blood pressure was defined as systolic blood pressure ≥ 140 mmHg and/or diastolic blood pressure ≥ 90 mmHg or known hypertensive on treatment. Venous blood sample (3 mL) was drawn from each patient in the morning after an overnight fast of eight to ten hours into gel separator tubes. The gel separator tubes were centrifuged at 3000 revolutions per minute for ten minutes and the serum separated and kept in plain separator tubes at a temperature of -20°C till it was time for analysis. The Automated Flexor Junior Chemistry Analyzer was used to analyze the samples. eGFR was computed using the serum creatinine in Modification of Diet in Renal Disease (MDRD) equation as indorsed by the National Kidney Disease Education Program (2015). eGFR < 60 mL/min/1.73 m^2^ shows renal dysfunction (chronic kidney disease) [23].

### 2.1. Statistical Analysis

Statistical analysis was performed using SPSS 22 software. Univariate analysis was done using chi-square or Fisher's exact test and Student's *t*-test. Multivariate binary logistic regression analysis was run for variables that were statistically significant in the univariate analysis. *p* < 0.05 was deemed statistically significant at two-tailed tests. Responses were shown using percentages and cross tabulations and presented in tables.

### 2.2. Ethical Considerations

Approval to conduct the study was given by the Committee on Human Research, Publications and Ethics of the Kwame Nkrumah University of Science and Technology (CHRPE/AP/228/15). Informed consent was obtained from participants after providing them with sufficient information about the study. Participants were also assured of the confidentiality of the information provided.

## 3. Results and Discussion

### 3.1. Results

A total of 100 diabetes patients were enrolled into the study. The sex distribution of the study participants was 31% males and 69% females with a mean age of 53.8 ± 13.8 years. Sociodemographic characteristics of the participants are shown in Table 1. The prevalence of diabetic foot ulcers (DFU) was 11%. Tables 2 and 3 show the general and biochemical characteristics in univariate analysis, respectively. In the univariate analysis, previous foot ulcer, foot deformity, impaired vision, serum creatinine, and eGFR were significantly associated with diabetic foot ulcer risk. In the multivariate analysis, previous foot ulcer and foot deformity were significant predictors. Diabetes patients with history of previous foot ulcer were 40.4 times more likely to develop foot ulcers than diabetes patients without history of previous foot ulcer. Diabetics with foot deformity were 14.4 times more likely to develop foot ulcers than diabetics without foot deformity. Impaired vision (*p* = 0.063), serum creatinine (*p* = 0.087), and eGFR (*p* = 0.937) could not maintain statistical significance in the multivariate logistic regression analysis (Table 4).

The prevalence of diabetic lower limb amputations (DLLA) was 3%. Foot deformity and serum urea were significantly associated with diabetic lower extremity amputation risk in the univariate analysis (Tables 5 and 6, respectively). Because of the low prevalence of DLLA (3%), multivariate logistic regression analysis was not done.

### 3.2. Discussions

In the present study, the prevalence of foot ulcers was 11%. This prevalence is higher than the prevalence reported in a retrospective study involving only type 2 diabetics aged >30 years in Ghana, where the prevalence was 3.8% [19]. Similarly, the prevalence in the current study was also higher than prevalences reported in other countries including Egypt, Kenya, Jordan, and Saudi Arabia where the prevalences were found to be 1.2%, 4.6%, 2.05%, and 3.3%, respectively [24–27]. However, the finding of this study is comparable to studies done in India, Ethiopia, and Tanzania, where the prevalences were found to be 14%, 14.8%, and 15%, respectively [28–30]. This wide variation in the prevalences of foot ulcers could be due to differences in subject characteristics and the methodology used. The high prevalence of foot ulcers reported in the present study may be due to the fact that the study was carried out in three tertiary hospitals that receive the largest referrals of patients in Ghana. Referral patients commonly present with a lot of complications including diabetes complications.

BMI was not identified as a risk factor for diabetic foot ulcer (DFU) or diabetic lower limb amputation (DLLA). Similarly, Kafrawya et al. [31] and Jung et al. [32] showed that BMI is not related to DFU and DLLA, respectively. In contrast, Pinzur et al. [33] and Sohn et al. [34] showed that BMI is correlated with DFU and DLLA, respectively, and may be attributed to the effect of body weight on plantar pressure, but evidence for this correlation is inconsistent [35].

Studies by Hellar and Mbembati [36] and Nyamu et al. [25] showed that hypertension is not a significant risk factor for diabetic foot ulcers. Also, hypertension was not identified as a risk factor for DLLA by Gürlek et al. [37] and Jung et al. [32] which confirms the finding of the present study. In contrast, Khan et al. [38] found a strong correlation between hypertension and DFU, and Lee et al. [39] also found a link between hypertension and DLLA. The differences in findings may be due to differences in the methodology used and population characteristics as confirmed by Bergqvist et al. [40].

The current study further identified smoking as an insignificant risk factor for DFU and DLLA. Similarly, Altenburg et al. [41] and Alder et al. [42] indicated that smoking is not a predictor of DFU and DLLA, respectively. Contrarily, other studies showed a correlation between smoking and diabetic foot ulcers as Musa and Ahmed [43] and Kafrawya et al. [31]. Smoking has been described theoretically as a cause of peripheral vascular disease in diabetics which often results in DFU [39] and thus DLLA. Smoking has been identified in a study not to be related to peripheral vascular disease [44], hence the finding of the present study.

Dyslipidemia has been identified to play a role in the etiology of peripheral vascular diseases, one of the main causes of DFU and DLLA, but this relationship is inconsistent, as dyslipidemia has also been reported not to be linked to diabetic peripheral vascular disease [44]. This may explain why the present study did not identify dyslipidemia as a risk factor for both DFU and DLLA. Similarly, Hellar and Mbembati [36] and Rajamani et al. [45] showed that dyslipidemia is not correlated to DFU and DLLA, respectively.

The duration of diabetes was not found to be related to either DFU or DLLA in the present study. In contrast, Al-Rubeaan et al. [27] revealed that DFU is correlated with diabetes duration. Similarly, Jbour et al. [46] found a correlation between diabetes duration and DLLA. Although longer diabetes duration has been linked to nerve damage (neuropathy), as described by the World Health Organization [47], this link has been proven to be very poor [48], thus justifies the finding of the present study. It has also been revealed in the current study that treatment modalities such diet, insulin, and oral hypoglycemic agents are not correlated with either DFU or DLLA. This finding is in line with that of Musa and Ahmed [43] who indicated that diet, insulin, and oral hypoglycemic agents are not correlated with DFU. Similarly, Yekta et al. [49] reported that diet or oral hyperglycemic agent treatment is not correlated with DLLA. Contrarily, a study by Shahi et al. [50] indicated that insulin use is significantly associated with DFU. Similarly, Krittiyawong et al. [51] showed that insulin use is correlated with DLLA. The differences in findings may be due to differences in the methodology used [40].

Theoretically, alcohol intake has been noted to cause nerve damage which can result in foot ulcers and amputations [52] but other studies reported no connection between nerve damage in diabetics and alcohol intake, thus suggesting an insignificant correlation between alcohol intake and DFU, and alcohol intake and DLLA as identified by the present study [30, 53, 54]. On the other hand, a study showed that alcohol use increases the risk of foot ulcers in diabetics [40].

Previous history of foot ulcer was found to be a predictor of diabetic foot ulcers in the present study. The finding of the present study has also been confirmed by Kafrawya et al. [31], Crawford et al. [55], and Abbott et al. [56]. After wound healing following ulceration, the plantar skin to that area usually become less strong to withstand repetitive stress and thus easily break down [57], hence the finding of the present study. Contrarily, other studies [30, 58] reported no link. However, previous history of foot ulcers was not related to DLLA risk in this study. Similarly, Siddiqui et al. [59] and Monteiro et al. [60] showed that previous history of foot ulcer does not increase DLLA risk. Regarding previous history of amputation, it was not found to be significantly associated with foot ulcer risk and DLLA in the present study. On the contrary, Kafrawya et al. [31] showed a link between previous history of amputation and diabetic foot ulcers. Rajamani et al. [45] showed that previous history of amputation increases DLLA risk. His finding might have differed from that of the current study possibly because it was limited to type 2 diabetics as difference in study characteristics can lead to different outcomes [61].

Some previous studies [62–64] revealed that increasing age is correlated with increased risk of foot ulcers in diabetics. These findings may be supporting the effect of increasing age on wound healing duration in diabetics [65]. In contrast, this relationship was not supported by Kafrawya et al. [31]. Similarly, the present study showed no correlation between age and foot ulcers as well as DLLA as reported in other studies [37, 60, 66]. In contrast, other studies [21, 32, 64] revealed a correlation between age and lower extremity amputations. The study designs and population characteristics differed among the studies and this may account for the difference in findings [67].

Foot deformities were associated with DLLA risk in the present study. The finding of this study is in consonance with previous studies [24, 31, 38, 56]. This may be as a result of the role of foot deformities in the etiology of DFU.

The results of this study will be useful for the management of diabetic foot ulcers and lower extremity amputations to enhance the quality of life of diabetes patients as well as contribute to further research and to policy formulation and implementation. However, this study has some limitations that are worth mentioning. The cross-sectional nature of the study does not provide a good basis for establishing causality as both exposure and outcome were assessed simultaneously. Moreover, the presence of impaired vision was based on self-reported diagnosis and this could have affected the outcome of the study.

## 4. Conclusions

This study assessed the prevalence and determinants of DFU and DLLA among diabetics attending hospitals in Ghana. The study showed that the prevalences of DFU and DLLA are high among diabetes patients. Foot deformities and previous history of foot ulcers are determinants of diabetic foot ulcers. Foot deformity and serum urea are associated with diabetic lower extremity amputations.

## Figures and Tables

**Table 1:** Sociodemographic characteristics of study participants.

Characteristics	Frequency (%/mean ± SD)
Age (years)	53.8 ± 13.8
Age groups	
18-29	5 (5)
30-39	12 (12)
40-49	18 (18)
50-59	25 (25)
≥60	40 (40)
Sex	
Male	31 (31)
Female	69 (69)
Ethnicity	
Northerner	36 (36)
Ga/Adangbe	9 (9)
Ewe	5 (5)
Akan	50 (50)
Marital status	
Single	9 (9)
Married	83 (83)
Divorced	6 (6)
Widowed	2 (2)
Religion	
Muslim	36 (36)
Christian	64 (64)
Level of education	
Primary	3 (3)
JHS	26 (26)
SHS	14 (14)
Tertiary	14 (14)
Informal	2 (2)
None	41 (41)
Employment status	
Employed	20 (20)
Self-employed	62 (62)
Not employed	18 (18)

**Table 2:** Univariate analysis of risk factors associated with DFU.

Characteristic	*N* = 100	DFU *N* = 11 (%)	No DFU *N* = 89 (%)	*p* value
Age (mean ± SD)	53.8 ± 13.8	54.2 ± 9.2	53.8 ± 14.3	0.874
Diabetes duration (yrs)				
<5	36 (100)	4 (11.1)	32 (88.9)	0.261
5-10	32 (100)	3 (9.4)	29 (90.6)	
11-15	16 (100)	4 (25)	12 (75)	
16-20	11 (100)	0 (0.0)	11 (100)	
>20	5 (100)	0 (0.0)	5 (100)	
Diabetes treatment				
Diabetic diet	14 (100)	2 (14.3)	12 (85.7)	0.120
OHAs	54 (100)	4 (7.4)	50 (92.6)	
Insulin	18 (100)	1 (5.6)	17 (94.4)	
OHAs and insulin	14 (100)	4 (28.6)	10 (71.4)	
Previous foot ulcer				
Yes	17 (100)	8 (47.1)	9 (52.9)	≤0.001
No	83 (100)	3 (3.6)	80 (96.4)	
Previous amputation				
Yes	5 (100)	1 (20)	4 (80)	0.509
No	95 (100)	10 (10.5)	85 (89.5)	
Smoking				
Yes	1 (100)	0 (0.0)	1 (100)	0.732
No	99 (100)	11 (11.1)	88 (88.9)	
Alcohol intake				
Yes	9 (100)	0 (0.0)	9 (100)	0.269
No	91 (100)	11 (12.1)	80 (87.9)	
BMI				
Underweight	9 (100)	0 (0.0)	9 (100)	0.180
Normal	28 (100)	5 (17.9)	23 (82.1)	
Overweight	40 (100)	2 (5)	38 (95)	
Obese	23 (100)	4 (17.4)	19 (82.6)	
Foot deformity				
Present	6 (100)	3 (50)	3 (50)	0.002
Absent	94 (100)	8 (8.5)	86 (91.5)	
Impaired vision				
Present	44 (100)	8 (18.2)	36 (81.8)	0.042
Absent	56 (100)	3 (5.4)	53 (94.6)	
Hypertension				
Present	60 (100)	6 (10)	54 (90)	0.695
Absent	40 (100)	5 (12.5)	35 (87.5)	

**Table 3:** Univariate analysis of biochemical characteristics associated with DFU among study participants.

Characteristic	*N* = 100	DFU *N* = 11 (%)	No DFU *N* = 89 (%)	*p* value
Serum Cr. (*μ*mol/L)				
Abnormal (>120)	14 (100)	4 (28.6)	10 (71.4)	0.023
Normal (≤120)	86 (100)	7 (8.1)	79 (91.9)	
eGFR (mL/min/1.73 m^2^)				
Abnormal (<60)	27 (100)	6 (22.2)	21 (77.8)	0.029
Normal (≥60)	73 (100)	5 (6.8)	68 (93.2)	
Serum urea (mmol/L)				
Abnormal (>8.3)	3 (100)	1 (33.3)	2 (66.7)	0.209
Normal (≤8.3)	97 (100)	10 (10.3)	87 (89.7)	
Total Chol. (mmol/L)				
Abnormal (>6.5)	7 (100)	1 (14.3)	6 (85.7)	0.773
Normal (≤6.5)	93 (100)	10 (10.8)	83 (89.2)	
Triglycerides (mmol/L)				
Abnormal (>1.7)	32 (100)	4 (12.5)	28 (87.5)	0.742
Normal (≤1.7)	68 (100)	7 (10.3)	61 (89.7)	
HDL Chol. (mmol/L)				
Abnormal (<1.03)	22 (100)	1 (4.5)	22 (95.5)	0.273
Normal (≥1.03)	78 (100)	10 (12.8)	68 (87.2)	
LDL Chol. (mmol/L)				
Abnormal (>4.9)	3 (100)	0 (0.0)	3 (100)	0.536
Normal (≤4.9)	97 (100)	11 (11.3)	86 (97)	

**Table 4:** Multivariate analysis of factors associated with foot ulcers among study subjects.

Characteristic	aOR	95% CI	*p*
Previous foot ulcer			
No	1		
Yes	40.4	5.4-299.9	≤0.001
Foot deformity			
Absent	1		
Present	14.4	1.3-161.2	0.031
Impaired vision			
Absent	1		
Present	7.1	0.9-55.6	0.063
Serum Cr. (*μ*mol/L)			
Normal (≥60)	1		
Abnormal (<60)	10.1	0.7-144.5	0.087
eGFR (mL/min/1.73 m^2^)			
Abnormal (<60)	1		
Normal (≥60)	1.1	0.1-9.6	0.937

**Table 5:** Univariate analysis of risk factors associated with DLLA.

Characteristic	*N* = 100	DLLA *N* = 3 (%)	No DLLA *N* = 97 (%)	*p* value
Age (mean ± SD)	53.8 ± 13.6	56.3 ± 8.1	53.7 ± 13.9	0.643
Diabetes duration				
<5	36 (100)	1 (2.8)	35 (97.2)	0.897
5-10	32 (100)	1 (3.1)	31 (96.9)	
11-15	16 (100)	1 (6.2)	15 (93.8)	
16-20	11 (100)	0 (0.0)	11 (100)	
>20	5 (100)	0 (0.0)	5 (100)	
Diabetes treatment				
Diabetic diet	14 (100)	1 (7.1)	13 (92.9)	0.293
OHAs	54 (100)	0 (0.0)	54 (100)	
Insulin	18 (100)	1 (5.6)	17 (94.4)	
OHAs and insulin	14 (100)	1 (7.1)	13 (92.9)	
Previous foot ulcer				
Yes	17 (100)	1 (5.9)	16 (94.1)	0.444
No	83 (100)	2 (2.4)	81 (97.6)	
Previous amputation				
Yes	5 (100)	1 (20)	4 (80)	0.116
No	95 (100)	2 (2.1)	93 (97.9)	
Smoking				
Yes	1 (100)	0 (0.0)	1 (100)	0.860
No	99 (100)	3 (3)	97 (97)	
Alcohol intake				
Yes	9 (100)	0 (0.0)	9 (100)	0.580
No	91 (100)	3 (3.3)	88 (96.7)	
BMI				
Underweight	9 (100)	1 (11.1)	8 (88.9)	0.328
Normal	28 (100)	1 (3.6)	27 (96.4)	
Overweight	40 (100)	0 (0.0)	40 (100)	
Obese	23 (100)	1 (4.3)	22 (95.7)	
Foot deformity				
Present	6 (100)	1 (16.7)	5 (83.3)	0.043
Absent	94 (100)	2 (2.1)	92 (97.9)	
Impaired vision				
Present	14 (100)	0 (0.0)	14 (100)	0.119
Absent	56 (100)	3 (5.4)	53 (94.6)	
Hypertension				
Present	60 (100)	2 (3.3)	58 (96.7)	0.811
Absent	40 (100)	1 (2.5)	39 (97.5)	

**Table 6:** Univariate analysis of biochemical characteristics associated with lower extremity amputations among study subjects.

Characteristic	*N* = 100	DLLA *N* = 3 (%)	No DLLA *N* = 89 (%)	*p* value
Serum Cr. (*μ*mol/L)				
Abnormal (>120)	14 (100)	1 (7.1)	13 (85.7)	0.241
Normal (≤120)	86 (100)	2 (2.3)	84 (98.8)	
eGFR (mL/min/1.73 m^2^)				
Abnormal (<60)	27 (100)	2 (7.4)	72 (98.6)	0.116
Normal (≥60)	73 (100)	1 (1.4)	25 (92.6)	
Serum urea (mmol/L)				
Abnormal (>8.3)	97 (100)	1 (33.3)	2 (66.7)	0.002
Normal (≤8.3)	3 (100)	2 (2.1)	95 (97.9)	
Total Chol. (mmol/L)				
Abnormal (>6.5)	7 (100)	0 (0.0)	90 (96.8)	0.629
Normal (≤6.5)	93 (100)	3 (3.2)	7 (100)	
Triglycerides (mmol/L)				
Normal (≤1.7)	32 (100)	2 (6.2)	30 (93.8)	0.191
Abnormal (>1.7)	68 (100)	1 (3)	67 (97)	
HDL Chol. (mmol/L)				
Abnormal (<1.03)	22 (100)	0 (0.0)	22 (100)	0.350
Normal (≥1.03)	78 (100)	3 (3.8)	75 (96.2)	
LDL Chol. (mmol/L)				
Abnormal (>4.9)	3 (100)	0 (0.0)	3 (100)	0.757
Normal (≤4.9)	97 (100)	3 (3.1)	94 (96.9)	

## Data Availability

The data used to support the findings of this study are available from the corresponding author upon request.
